# Cross-Cultural Communication on Social Media: Review From the Perspective of Cultural Psychology and Neuroscience

**DOI:** 10.3389/fpsyg.2022.858900

**Published:** 2022-03-08

**Authors:** Di Yuna, Liu Xiaokun, Li Jianing, Han Lu

**Affiliations:** ^1^School of International Economics and Management, Beijing Technology and Business University, Beijing, China; ^2^School of Economics, Beijing Technology and Business University, Beijing, China; ^3^Institute of the Americas, University College London, London, United Kingdom

**Keywords:** cross-culture communication, social media, cultural psychology, neuroscience, cultural neuropsychology, social neuroscience

## Abstract

**Introduction:**

In recent years, with the popularity of many social media platforms worldwide, the role of “virtual social network platforms” in the field of cross-cultural communication has become increasingly important. Scholars in psychology and neuroscience, and cross-disciplines, are attracted to research on the motivation, mechanisms, and effects of communication on social media across cultures.

**Methods and Analysis:**

This paper collects the co-citation of keywords in “cultural psychology,” “cross-culture communication,” “neuroscience,” and “social media” from the database of web of science and analyzes the hotspots of the literature in word cloud.

**Results:**

Based on our inclusion criteria, 85 relevant studies were extracted from a database of 842 papers. There were 44 articles on cultural communication on social media, of which 26 were from the perspective of psychology and five from the perspective of neuroscience. There are 27 articles that focus on the integration of psychology and neuroscience, but only a few are related to cross-cultural communication on social media.

**Conclusion:**

Scholars have mainly studied the reasons and implications of cultural communication on social media from the perspectives of cultural psychology and neuroscience separately. Keywords “culture” and “social media” generate more links in the hot map, and a large number of keywords of cultural psychology and neuroscience also gather in the hot map, which reflects the trend of integration in academic research. While cultural characteristics have changed with the development of new media and virtual communities, more research is needed to integrate the disciplines of culture, psychology, and neuroscience.

## Introduction

Cross-cultural communication refers to communication and interaction among different cultures, involving information dissemination and interpersonal communication as well as the flow, sharing, infiltration, and transfer of various cultural elements in the world (Carey, 2009; Del Giudice et al., 2016). With more than half of the world’s population using social media, such as Facebook, Twitter, and WeChat, communication across culture has become smoother and more frequently (Boamah, 2018; Chin et al., 2021). Subsequently, cultural exchanges, collisions, conflicts, and integration among various nationalities, races, and countries on these platforms have become obvious, and related research articles by scholars in different disciplines have increased (Papa et al., 2020). In traditional cross-cultural research, experts often divide different cultures based on their boundaries, such as countries, races, languages, and so on. However, with the development of digitalization, new cultural relationships have been formed both within and outside geopolitical boundaries, and new understanding and theories are needed to explain the motivation, process, and implications of cross-cultural communications in the digital era (Chin et al., 2020). Research in this field is an emerging area, and scholars are studying from different perspectives (Xu et al., 2016; Santoro et al., 2021). Cultural psychology and neuroscience are two main base theories, and they show a trend of integration, such as cultural neuroscience and cultural neuropsychology. In this case, it is important to highlight the important achievements of this field and identify potential research gaps to provide potential directions for further research. This review aims to provide an overview of cross-cultural communication research from the perspective of cultural psychology and neuroscience and identify the integrating trend and potential directions.

## Method and Source

We used the Web of Science (WoS) database to select relevant articles published between January 2010 and December 2021. The following inclusion criteria were used:

1. The document types should be articles rather than proceedings papers or book reviews. And the articles should be included in the Web of Science Core Collection.
2. When searching for articles, the topic should include at least two keywords: “cultural psychology,” “neuroscience,” “social media.”
3. Articles must be published after 2010 to ensure the content of the literature is forward.
4. This study should investigate the integration of cultural psychology and neuroscience or explore cultural issues in social media from the perspective of cultural psychology or neuroscience. The content could be cultural conflict and integration on social platforms, explanations of cultural conflict and integration on social platforms, or integration of neuroscience and cultural psychology.

**Figure 1:**
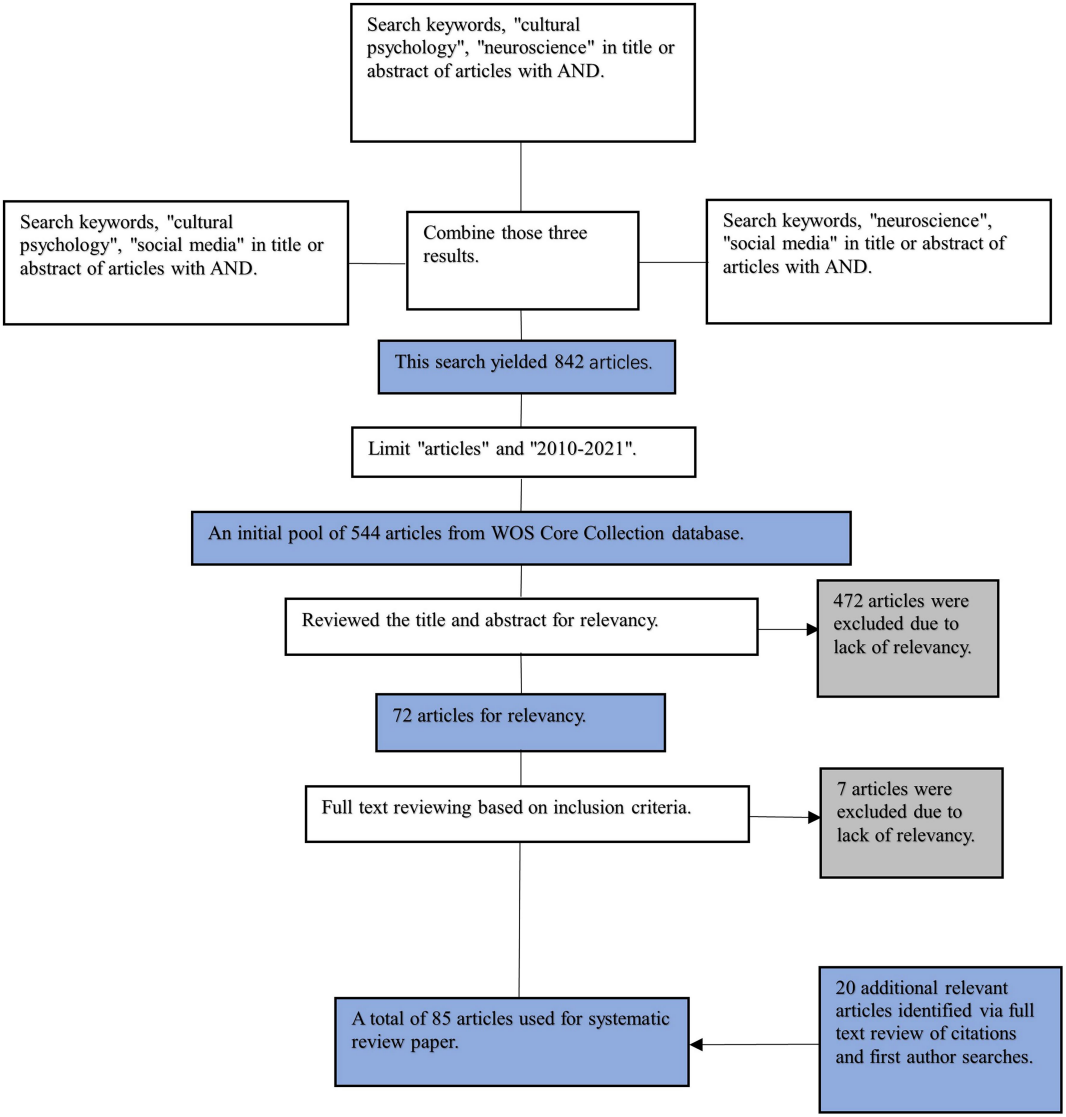
Schematic representation of literature search and selection procedure.

Based on the above inclusion criteria, 85 relevant studies were searched, analyzed, and evaluated. These documents were identified according to the procedure illustrated in Figure 1. The following combinations of keywords were used: (cultural psychology AND social media), (neuroscience AND social media), (cultural psychology AND neuroscience), [social media AND (cross-cultural communication OR cultural conflict OR cultural integration)], and (neuroscience, cultural psychology, and cross-cultural). The number of studies was further reduced by limiting the document type and time range. Consequently, we obtained an initial pool of 544 articles. To ensure the relevance of the literature in the initial pool, we reviewed the titles and abstracts of these articles. Articles targeting pure neuroscience and information technology were excluded and 72 articles were retained. We selected 65 articles after reviewing the full text. For most papers excluded from the initial pool, cultural issues on social media were not the main topic but digital media or culture itself. The most typical example of irrelevant articles was that culture or cultural psychology was only briefly mentioned in the abstracts. Moreover, 20 additional relevant articles were identified *via* full-text review of citations and first author searches. Using the above steps, 85 articles were selected for the literature review.

## Results

### Overview of Selected Articles

**Figure 2:**
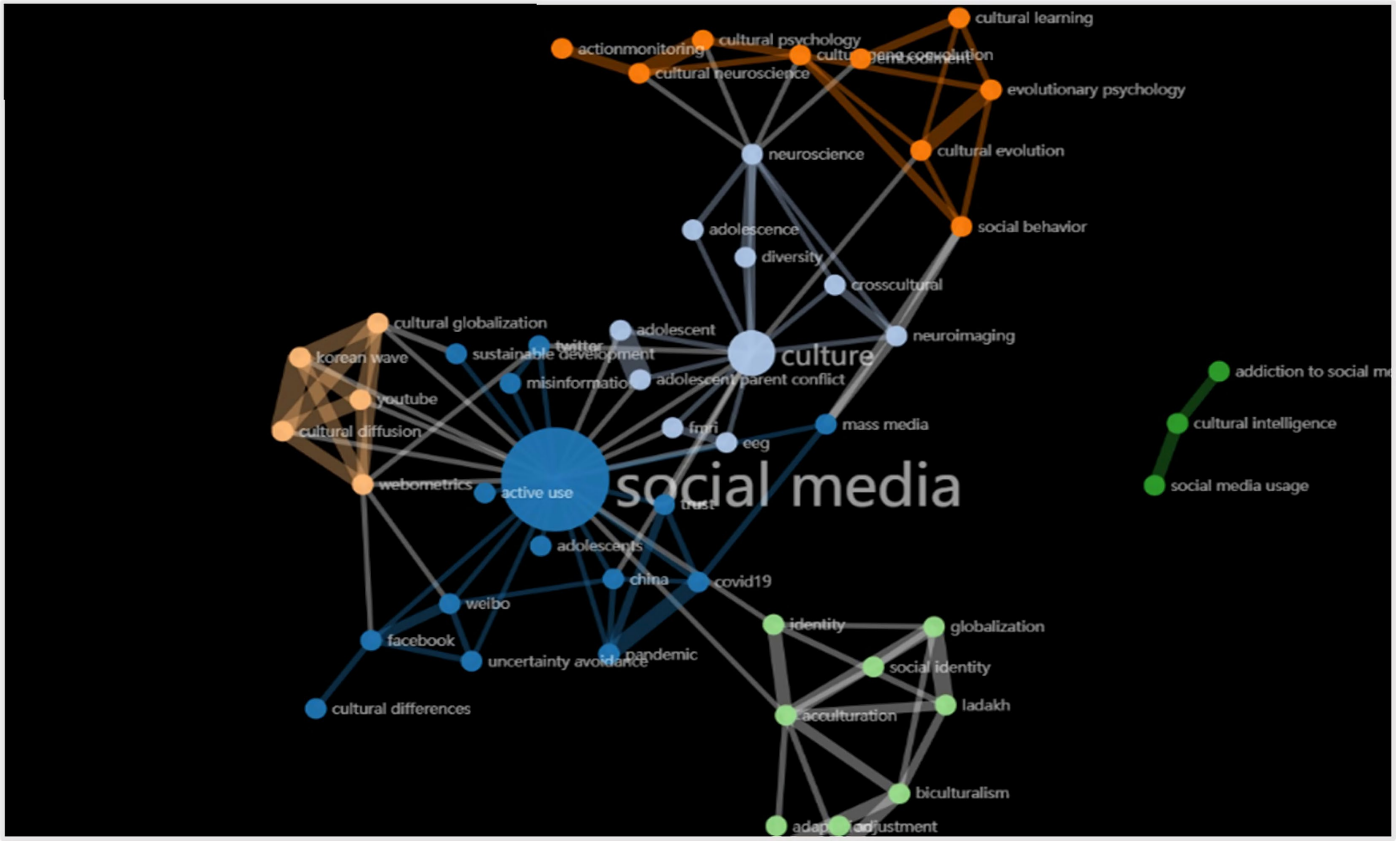
Keywords knowledge graph.

Here, frequency refers to the percentage of occurrences of an item in the total number of studies. The keywords “acculturation,” “cultural evolution” occurred frequently together with “social media,” “culture,” and “neuroscience.” This is as expected because psychologists and economists have long known that human decision-making is influenced by the behavior of others and that public information could improve acculturation and lead to cultural evolution. The popularity of social media clearly gives public information an opportunity to spread widely, which has caused an increase in research on the cross-cultural communication of social media. In the last decade, the link between cultural issues and social media research has grown. This is reflected in the knowledge graph (Figure 2). Keywords “culture” and “social media” generate lots of links with “social media” and “mass media,” which is shown in blue node groups and white node groups. “Social media” and “cultural globalization,” “biculturalism,” “acculturation” also form node convergences. The integration of neuroscience and cultural psychology is also represented in Figure 2 as an orange node group. These integration trends can also be verified in the time dimension. As time passes, keyword frequencies have changed from a single component of “social media” or “culture” to a multi-component of “social media,” “culture,” “acculturation,” “neuroscience,” “cultural evolution.” The frequency of all keywords is presented through the overall word cloud.

We identified three different research topics from the 85 selected articles: cross-cultural communication on social platforms, explanation of cultural conflict and integration on social platforms, and the integration of neuroscience and cultural psychology. Existing literature has analyzed and studied the interaction between cross-cultural users, enterprises, and countries on social media. For instance, some scholars have found that social media play a significant role in negotiating and managing the identity of transient migrants relating to the home and host culture during the acculturation process (Cleveland, 2016; Yau et al., 2019). Social media usage by expatriates also promotes cultural identity and creativity (Hu et al., 2020). In addition to the discussion of existing phenomena, many articles have discussed the causes of social media cultural transmission. A new research field, cultural neuroscience, indicates the integration of neuroscience and cultural psychology. These issues are reviewed in the following sections.

There were 44 articles on cultural communication on social media, which accounted for 51.76% of the 85 selected papers. Among these, there were 26 studies on cultural communication on social media from the perspective of psychology, five articles from the perspective of neurology, four articles about enterprises using social media for cross-cultural operations, and nine articles about how governments use social media for cross-cultural communication. Although there are 27 articles that discuss the trend of integration of psychology and neuroscience, few use integrated methods to analyze the behavior of cross-cultural communication.

### From Perspective of Cultural Psychology

Cultural psychology researchers have focused on why information is shared. Some scholars have divided the reasons into individual and network levels (Wang et al., 2021). Studies have explored information sharing within a specific domain, such as health information and news dissemination (Hodgson, 2018; Li et al., 2018; Wang and Chin, 2020). Cultural psychology provides a rich explanation for the factors that influence cultural communication. Cultural background affects the process of cultural communication, such as self-construal, which the host country may alter it (Huang and Park, 2013; Thomas et al., 2019). This may influence communication behaviors, such as people’s intention to use social media applications, attitudes toward social capital, social media commerce, and sharing behavior itself (Chu and Choi, 2010; Han and Kim, 2018; Li et al., 2018).

Factors other than culture cannot be ignored: public broadcast firms and fans promote communication, controversial comments may draw more attention, the sociality of the social media capsule expands the scope of information communication, and how news is portrayed has changed (Meza and Park, 2014; Jin and Yoon, 2016; Hodgson, 2018). Demographic factors, such as sex and age, are not ineffective (Xu et al., 2015). The experiential aspects have also been noted (Wang et al., 2021). Scholars have also noted the importance of cultural intelligence (Hu et al., 2017).

The topic that researchers are most interested in is the relationship between society and individuals. Many studies have focused on the influence of collectivist and individualist cultures, such as social media users’ activity differences, attentional tendencies, and self-concept (Chu and Choi, 2010; Thomas et al., 2019). There are some other interesting topics, such as the relationship among multicultural experiences, cultural intelligence, and creativity, the evaluation of the validity of the two measures, the changing status of crucial elements in the social system, and the government effect in risk communication (Hu et al., 2017; Ji and Bates, 2020). Extending to the practical level, mobile device application usability and social media commerce were evaluated (Hoehle et al., 2015; Han and Kim, 2018).

At the methodological level, researchers have bridged the gap between reality and online behaviors, and the feasibility of social media dataset analysis has been proven (Huang and Park, 2013; Thomas et al., 2019). Some new concepts have been examined and some models have been developed (Hoehle et al., 2015; Li et al., 2018). The most common method is to quantify questionnaire information (Chu and Choi, 2010; Hu et al., 2017; Han and Kim, 2018; Li et al., 2018; Wang et al., 2021). The online survey accounted for a large proportion of respondents. Exploratory factor analysis (EFA) is used to evaluate other measures (Ji and Bates, 2020). Researchers are particularly interested in the metric approach (Meza and Park, 2014). Some combine other methods, such as profile and social network analyses (Xu et al., 2015). Scholars have used qualitative research to obtain detailed feedback from respondents (Jin and Yoon, 2016; Hodgson, 2018). Content analysis was also used (Yang and Xu, 2018).

### From Perspective of Neuroscience

Neuroscientific explanations focus on understanding the mechanisms of cultural conflict and integration. Neuroscience researchers are concerned about the effects of the brain on cultural communication and the possible consequences of cultural communication on human behavior and rely on the study of the brain as a tool. Neuroscience can be used to study how people behave in reality. Given the similarity between offline and online behaviors, neuroscience can study online behaviors and link them to cultural communication (Meshi et al., 2015). Cross-sectional and longitudinal studies, both inside and outside the laboratory, have become the subject of neuroscience studies. One example of long-term studies outside the laboratory is the study of natural Facebook behavior that was recorded for weeks (Montag et al., 2017).

Motivation research is a well-documented topic. The reason for using social media, motivation to share information, and neural factors related to sharing behavior have been discussed (Fischer et al., 2018). Many scholars have connected motivation with social life based on the inseparable relationship between online behaviors and social life. Some academics hope to provide predictions of real life, such as forecasting marketing results, while some warned of the risks, in which tremendous attention has been paid to the situation of adolescents (Motoki et al., 2020). They are susceptible to acceptance and rejection (Crone and Konijn, 2018). Behavioral addiction and peer influence in the context of risky behaviors also lead to public concern (Meshi et al., 2015; Sherman et al., 2018).

On a practical level, neuroscience studies have made predictions possible through the findings of activity in brain regions linked to mentalizing (Motoki et al., 2020). Judgments of social behavior are also warranted, and peer endorsement is a consideration (Sherman et al., 2018). Thus, the dangers of cultural communication can be alleviated.

At the methodological level, the feasibility of linking directly recorded variables to neuroscientific data has been proven, which provides a methodological basis for further studies linking neuroscience and cultural communication (Montag et al., 2017). Neuroscience researchers have shown a preference for magnetic resonance imaging (MRI) methods, which include functional and structural MRI scans (Montag et al., 2017; Sherman et al., 2018). Although some scholars have pointed out the shortcomings of MRI research and attempted to use the electroencephalographic (EEG) method, most scholars still use MRI and combine it with other methods, such as neuroimaging (Motoki et al., 2020). Despite the similarities in the methods used, there were differences in the scanned areas. Some researchers scan multiple regions, such as the ventral striatum (*VS*) and ventromedial prefrontal cortex (VMPFC), while others focus on analyzing the content of a single region, such as the nucleus accumbens (NAcc; Baek et al., 2017). Related characteristics have been discussed, such as theta amplitudes that affect information sharing (Fischer et al., 2018). Some inquire whether the different properties of brain regions can lead to different results (Montag et al., 2017).

### Integration of Neuroscience and Cultural Psychology

Of the 85 papers we selected, 27 discussed the integrated development of psychology and neuroscience, and the number of articles in this discipline increased. Cultural psychology has made remarkable progress in identifying various cultural traits that can influence human psychology and behavior on social media. Cultural neuroscience as a cross-subject of the rise in recent years, through the integration of psychology, anthropology, genetics, neuroscience, and other disciplines, explains the interaction of culture and the human brain, and how they jointly affect the neural mechanism of cognitive function. At an early stage, scholars presented the interactive dynamic evolutionary relationship between the brain and culture from multiple perspectives (Moffittet et al., 2006). However, with technological improvements in brain imaging, it is possible to solve and explore interactions between the human brain, psychology, and cultural networks using an empirical approach.

Cultural characteristics have dramatically changed during the last half-century with the development of new media and new virtual ways of communication (Kotik-Friedgut and Ardila, 2019). Existing research has shown that the neural resources of the brain are always adapted to the ever-increasing complexity and scale of social interaction to ensure that individuals are not marginalized by society (Dunbar and Shultz, 2007). The interaction between biological evolution and cultural inheritance is a process full of unknowns and variables. Therefore, research on the relationships between culture, psychology, and neuroscience will progress together.

At the methodological level, communication on social media by users from different backgrounds provides a new research environment and massive data for cross-disciplinary research. Big data on social media and AI technology can analyze not only the reactions, emotions, and expressions of an individual but also the relevant information of an ethnic group or a cultural group. A number of neurological and psychological studies are beginning to leverage AI and social media data, and the two disciplines are intertwined with each other (Pang, 2020; Wang et al., 2021). This quantitative analysis also helps enterprises and government departments to understand and affect cultural conflicts and integration (Bond and Goldstein, 2015).

## Discussion

### Different Schools of Thoughts

Social media provides platforms for communication and facilitates communication across cultures; however, the specific content exchanged is considered from the perspective of cultural proximity. Although some scholars think that social media can significantly promote mutual acceptance and understanding across cultures, others have realized that digital platforms actually strengthen the recognition and identity of their respective cultures (Hopkins, 2009). To study the motivations, results, and implications of cross-cultural communication in virtual communities and conduct an empirical analysis, psychologists and neuroscientists provide their grounds and explanations.

### Current Research Gaps

Although there are many articles discussing the trend of integration of psychology and neuroscience, few of them use integrated methods to analyze the behavior and implications of cross-cultural communication, mainly on cultural evolution and social effects. There are both practical and theoretical needs to be addressed to promote deep integration. For example, both private and public departments urgently need to learn scientific strategies to avoid cultural conflicts and promote integration. Further, a systematic and legal theory is also needed for scholars to conduct research in the sensitive field, which may be related to privacy protection and related issues.

### Potential Future Development

For the research object, the classification of culture in emerging research is general, while with the development of big data methods on social media, cross-cultural communication among more detailed groups will be a potential direction. For the research framework, although cultural neuroscience is already a multidisciplinary topic, the ternary interaction among the brain, psychology, and culture in a virtual community will be very important. For the research method, brain imaging technology-related data and social media data may cause issues, such as privacy protection, personal security, informed consent, and individual autonomy. These legal and ethical issues require special attention in the development process of future research.

## Conclusion

Cross-cultural communication research in the digital era not only needs to respond to urgent practical needs to provide scientific strategies to solve cultural differences and cultural conflicts, but also to promote the emergence of more vigorous theoretical frameworks and methods. Existing articles have mainly studied the reasons and implications of cultural communication on social media from the perspectives of cultural psychology and neuroscience separately. The CiteSpace-based hot topic map also shows the clustering trend of keywords related to cultural psychology and neuroscience, reflecting the intersection of the two fields. At the same time, there are many links between the two keyword nodes of “culture” and “social media,” which indicates that there is no lack of studies on cultural communication on social media from the perspective of cultural psychology. While cultural characteristics have changed with the development of new media and big data and related technologies have improved significantly, more research is needed to integrate the disciplines of culture, psychology, and neuroscience both in theory and methods.

## Author Contributions

All authors listed have made a substantial, direct, and intellectual contribution to the work and approved it for publication.

## Funding

This paper was funded by the Beijing Social Science Fund, China (Project No. 21JCC060).

## Conflict of Interest

The authors declare that the research was conducted in the absence of any commercial or financial relationships that could be construed as a potential conflict of interest.

## Publisher’s Note

All claims expressed in this article are solely those of the authors and do not necessarily represent those of their affiliated organizations, or those of the publisher, the editors and the reviewers. Any product that may be evaluated in this article, or claim that may be made by its manufacturer, is not guaranteed or endorsed by the publisher.
